# The 4^th^ National Neurology Forum | Interview with Associate Professor Cătălina Crișan

**DOI:** 10.25122/jml-2026-1012

**Published:** 2026-07

**Authors:** Stefana-Andrada Dobran, Alexandra Gherman

**Affiliations:** 1 RoNeuro Institute for Neurological Research and Diagnostic, Cluj-Napoca, Romania

Interviewee: Associate Professor Cătălina Crișan

Interviewer: Ms. Ștefana-Andrada Dobran

Cătălina A. Crișan is Associate Professor at Iuliu Hațieganu University of Medicine and Pharmacy in Cluj-Napoca and a senior psychiatrist at the Emergency County Hospital Cluj-Napoca.

Her clinical and research work focuses on psychiatric disorders, including the evaluation of disease awareness and coping mechanisms used by patients and the general population in crisis situations, as well as forensic psychiatry and the evaluation of psychiatric symptoms in neurodegenerative disorders, particularly Huntington’s disease and neurotraumatic brain injury. Her expertise in psychiatry includes specialized training in psychosis and mood disorders, as well as schizophrenia. Assoc. Prof. Cătălina Crișan is a member of the Commission of Psychiatric Forensic Expertise and of the Huntington’s disease service in Romania, where she is actively involved in the evaluation of patients and families affected by the condition.

Her academic work includes more than 55 ISI-indexed articles, 28 book chapters and six books in the field of psychiatry.

S.D.: Hello, Assoc. Prof. Cătălina Crișan, and welcome to the fourth edition of the National Neurology Forum!

**C.C.:** Hello. Thank you very much for the invitation.

S.D.: How do you see the role of the Forum in facilitating dialogue between specialties, in the context of the neurotraumatology workshop?


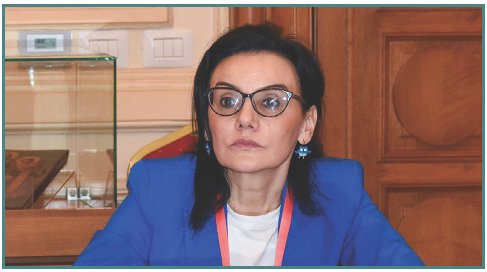



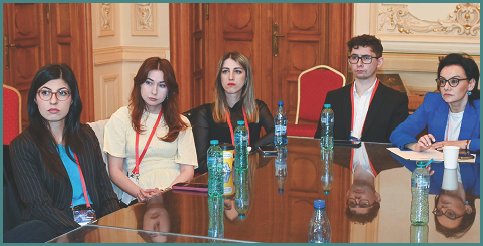


**C.C.:** First of all, I would like to thank Professor Dafin Mureșanu for inviting me to this Forum. This is my first time participating, and I am extremely impressed by the range of specialties [represented] and by the colleagues with outstanding expertise who have come here.

I think the only appropriate way to treat a patient with a neurotrauma is as part from a multidisciplinary team. In this regard, this Forum plays an important role in bringing together specialists from different medical fields so that they can approach the patient with neurotrauma as effectively and appropriately as possible.

S.D.: What are the main challenges in the multidisciplinary approach to patients with traumatic brain injuries, taking into account your specialty?

**C.C.:** The patient with neurotrauma experiences a drama – first of all, at the moment of impact, but also throughout subsequent recovery. For some patients, recovery is possible thanks to the coordinated care provided by a multidisciplinary team. Everyone has their place from the moment they take over the patient. Emergency doctors and paramedics are first at the scene of the accident and take the necessary emergency measures to stabilize the patient, intubate if necessary, monitor the patient, and maintain vital functions. Then, the patient is transferred to the intensive care unit, where our ICU colleagues perform extraordinary work, providing care during the critical initial moments and throughout the following days. Unfortunately, some patients require prolonged intensive care.

If necessary, of course, the neurosurgeon also has an important role, particularly when neurosurgical intervention is required. From the ICU or Neurosurgery, the patient will most often be transferred to a Neurology and/or Psychiatry department.

My role as a psychiatrist is to manage acute and delayed complication of a neurotraumatic event. In the acute phase, when the patient is in the intensive care unit or in the neurosurgery department, he can develop confusional syndrome (delirium) or psychomotor agitation. These syndromes need to be recognized and properly managed with different methods (non-pharmaceutical or with different psychotropics).

Another important role for a psychiatrist is to provide support during very difficult moments — for example, if the patient with neurotrauma loses one or several family members, or loses a part of his body. As psychiatrists, we can help patients manage the various emotional reactions they may experience (anxiety, depression, acute reaction to stress, grief, memory loss).

I also find the third role in the recovery process, during neurorehabilitation, which can take a very long time and is done with the help of colleagues from the rehabilitation specialty. During this phase, the patient can experience depressive episodes, post-traumatic stress disorder, and cognitive problems (mild cognitive impairment or even posttraumatic dementia).

To conclude, I see the role of our specialty from a longitudinal perspective—from the acute phase of the traumatic event throughout the entire recovery process.

S.D.: What do you consider to be the priority directions for the development of neuropsychiatric rehabilitation in Romania in the coming years?

**C.C.:** First of all, I think the key is to be a part from a multidisciplinary team–every specialist has a role in this team: the intensive care specialist, the neurosurgeon, the neurologist, the neurorehabilitation specialist, the psychiatrist, and the psychologist.

As for the directions, first of all, there should be a proper assessment of the psychiatric disorders which may occur in patients with neurotrauma, followed by their early recognition and appropriate management. As I was saying, the most common ones, even in the intensive care unit, are delirium (confusional syndrome), anxiety disorders, depressive disorders, post-traumatic stress disorder and cognitive disorders. The psychiatric treatment needs to take into consideration the treatment indicated by other specialists (neurosurgeons, neurologists, intensive care doctors).


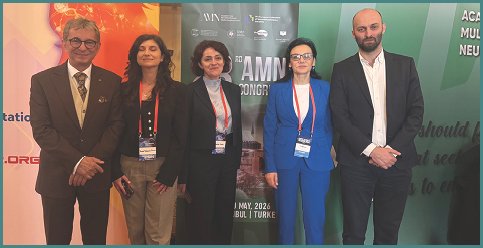


On the other hand, I think that cognitive rehabilitation programs are very important. There are cognitive remediation programs that use specific exercises targeting different cognitive functions, such as attention, attention, memory, or executive functions.

I think the psychological support is also very important - many patients can develop anxiety, depression, stress, sleep problems after such a major and impactful event. Patients often feel “the loss of self” before this event. This loss is experienced not only by the patient, but also by his family members. And this is where the psychologist plays an important role.

I would also emphasize the importance of family support. Perhaps we could consider support groups, similar to those organized by associations for patients with bipolar disorder, schizophrenia, Parkinson’s disease, or stroke, as well as support group for the patients' families. We could also work towards establishing an association for patients with neurotrauma and their families.

Earlier, during the FOCUS GROUP, I was talking about something that I believe is related to the individual nature of each person. One situation is if something acute happened to you and you manage to overcome it; a completely different situation is if the patient has serious sequelae, both physical and mental after a neurotraumatic event. The rehabilitation process can take months or even years. The patient needs a certain level of resilience and good coping mechanisms throughout this process.

If you lost your career—for example, you were a ballerina, you lost your legs, you can no longer dance ballet. Or maybe you were a lawyer and you developed an extremely serious cognitive disorder, you can no longer practice; or you were a high-performance athelte and you lost a leg. To have the strength to reinvent yourself and find a new path is not very easy.

As I learned in psychotherapy, there is always a positive side to everything that happens to you. Even in a dramatic event, we can find a positive and a negative part in the same time. This is called *positum*. You may realize that, even through a tragedy like this, you can find another path—one that perhaps was destined for you.

